# Treated Wastewater Effluent as a Source of Microbial Pollution of Surface Water Resources

**DOI:** 10.3390/ijerph110100249

**Published:** 2013-12-23

**Authors:** Shalinee Naidoo, Ademola O. Olaniran

**Affiliations:** Discipline of Microbiology, School of Life Sciences, College of Agriculture, Engineering and Science, University of KwaZulu-Natal, Private Bag X541 001, Westville Campus, Durban 4000, South Africa; E-Mail: shalinee23@gmail.com

**Keywords:** sanitation, treated wastewater, effluent disposal, microbial indicators, guidelines

## Abstract

Since 1990, more than 1.8 billion people have gained access to potable water and improved sanitation worldwide. Whilst this represents a vital step towards improving global health and well-being, accelerated population growth coupled with rapid urbanization has further strained existing water supplies. Whilst South Africa aims at spending 0.5% of its GDP on improving sanitation, additional factors such as hydrological variability and growing agricultural needs have further increased dependence on this finite resource. Increasing pressure on existing wastewater treatment plants has led to the discharge of inadequately treated effluent, reinforcing the need to improve and adopt more stringent methods for monitoring discharged effluent and surrounding water sources. This review provides an overview of the relative efficiencies of the different steps involved in wastewater treatment as well as the commonly detected microbial indicators with their associated health implications. In addition, it highlights the need to enforce more stringent measures to ensure compliance of treated effluent quality to the existing guidelines.

## 1. Introduction

Safe drinking water and proper sanitation have constantly been recognized as indispensable factors to sustain life. Nevertheless, despite remarkable global progress to improve access to drinking water facilities, currently there are 884 million and an additional 2.5 billion people lacking improved water sources and sanitation respectively [1]. This crisis is further compounded by factors such as increasing poverty, accelerated population growth and rapid urbanization coupled with hydrological variability and climate change. These socio-economic and environmental factors place even further stress on the deteriorating water and sanitation infrastructure, more so in developing regions, where billions are still at risk of Water, Sanitation and Hygiene (WaSH) related diseases. Despite meeting the Millennium Development Goals regarding access to potable water, the depletion of existing finite water resources still continues to be a major problem, with projections that approximately 605 million people will still lack access to improved drinking water by 2015 [1,2]. In addition, lack of access to potable water is estimated to cost countries between 1%–7% of their annual GDP, with slow water and sanitation-related progress further impeding national economic growth [1,3]. This together with the above named factors serve as the major driving force behind the increased use of wastewater, surrounding surface water and grey water for various recreational, agricultural and aquaculture activities [4].

Reliable wastewater treatment systems serve as a good indication of the level of development within a municipality as well as community health, with the degree and quality of wastewater determining the impact of these treatment plants on surrounding water sources into which it is released [5]. Over the last few years, the quantity of municipal wastewater produced has drastically increased due to the constant increase in population numbers together with an increased dependence on diminishing water resources. This coupled with the discharge of inefficiently treated wastewater into surrounding surface water sources serve as a direct threat, not only to the macro- and microflora and fauna present, but also to the provision of good quality water required for all socio-economic functions [2]. Thus the constant monitoring of the operational status of existing wastewater treatment plants (WWTPs) as well as increasing emphasis on environmental and water resource health has become key factors in determining the quantity and quality of wastewater generated by respective municipalities. This review highlights the importance of adequate wastewater management as one of the key steps in to protecting and ensuring the supply of safe drinking water and maintenance of good public health. In addition, it highlights the inefficiency of traditional wastewater practices as well as consequences of inadequately treated effluent discharge on the environment and human health. 

## 2. Sources of Domestic and Industrial Wastewater

Wastewater is defined as any storm water runoff, as well as industrial, domestic or commercial sewage or any combination thereof carried by water. The type and volume of wastewater generated is determined by both, population numbers and the combination of surrounding domestic, recreational and industrial activities, all of which affect discharge patterns as well as the chemical status of the treated effluent [6]. In order to set up an efficient waste management system, proper identification and characterization of the influent entering a wastewater treatment plant is essential [7]. This is based on the physical, chemical and biological characteristics of the influent; the immediate and downstream effect on the surrounding environment into which the wastewater will be discharged as well as the currently laid out environmental and discharge standards.

Four main types of wastewater have been identified, namely domestic, industrial, agricultural and urban. Urban wastewater is defined as a combination of domestic and industrial wastewater as well as surrounding sewage infiltration and rain water whilst agricultural wastewater consists of wastewater generated through processes from surrounding farms, agricultural activities and sometimes contaminated groundwater [8]. Generally, the focus is mainly on domestic and industrial sewage as a source of plant influent and contamination, however agricultural runoff is now becoming increasingly important due to the high quantities of pesticides and fertilizers being used, ultimately contributing to surface water eutrophication [5]. Domestic wastewater is defined as sewage which generally consists of black water composed of fecal matter (human and animal wastes) together with grey water sources composed of various wastewater constituents. These components originate from a range of household activities (washing and bathing) with each forming approximately 32.5% and 67.5% of domestic sewage respectively [9]. Initially, this water is used for drinking, food preparation, hot water systems, bathing, personal hygiene, washing and gardening and may ultimately form part of the domestic wastewater being excreted into the environment [10]. Within a household individual domestic wastewater streams all contribute different amounts to the overall nutrient and element load comprising the discharged effluent. Industrial wastewater however, is defined as sewage consisting of industrial wastes such as pulp, paper, petrochemical runoff as well as various chemicals, salts and acids. These sources vary widely in composition and often require special tertiary treatments in order to comply with discharge regulations. The composition of industrial wastewater varies based on the type of surrounding industry together with respective contaminant and pollutant composition with general classification into inorganic and organic industrial wastewater [11]. 

## 3. Impact of Improperly Treated Wastewater Effluent

### 3.1. Effect on the Environment, Micro- and Macrofauna

The biggest concern associated with microbial pollution is the risk of human and livestock related illnesses after exposure to contaminated water sources. Often the discharge of improperly treated effluent from WWTPs results in the deposition of large amounts of organic matter and nutrients which have major detrimental effects on the health of these surrounding environments as well as micro- and macro-fauna present. Excessive nutrient loading can lead to eutrophication and temporary oxygen deficiencies that ultimately alter the energy relationship and water balance, disrupting biotic community structure and function. Excessively turbid effluent discharge can also result in the deposition of sand and grit into the aquatic system, disrupting sediment characteristics and hindering natural water flows [12]. In addition, the overall hydrological and physicochemical environment is often affected due to the discharge of improperly treated effluent with many of the micro- and macro-fauna within these water bodies exhibiting distinct physiological tolerance levels. Disturbances to the overall environment can severely affect those intolerant individuals either in the form of adverse behavioural characteristics or more severely in the form of death. Often death decreases a large degree of resource competition and predation within the environment thereby resulting in the proliferation of tolerant organisms. This ultimately causes an imbalance amongst the group of organisms present and the overall alterations to the surrounding environment in the form of nutrient modifications, light and oxygen content, food sources as well as habitat loss [13]. Furthermore, the deposition of excessive nutrients leads to profuse plant growth along river banks which in certain cases may be visually pleasing but can serve as an additional health hazard due to entanglement and poor visibility. Benthic microbial and algal growth may also cause rock and wood surfaces to become slippery, posing a threat to human safety [12]. 

### 3.2. Effect on Human Health

Communities situated downstream or near to municipal sewage outfalls or contaminated water sources are at the highest risk of illness due to increased microbial pathogens and deteriorating physico-chemical parameters [12]. Often the discharge of extremely turbid effluent in conjunction with dense algal blooms results in poor visibility within these water bodies thus creating dangerous situations for recreational users. In addition, water bodies used for full contact recreational activities may serve as a source of various infectious diseases which may be contracted either by ingestion of contaminated water or through full body contact [10]. However, depending on the type of waterborne disease and on the physical health of the individual concerned, the person may either recover completely or suffer permanently from the resultant disease. In addition, a variety of skin and ear infections may arise as a result of contaminated water coming into contact with broken skin or penetration of the ear. Furthermore, discharge of improperly treated effluent often results in an increased number of bacterial, viral and protozoan pathogens which may result in a range of waterborne related diseases such as giardiasis and gastroenteritis [14]. A number of indirect health hazards such as chemical contaminants, disease-transmitting organisms such as mosquitos and fresh water snails implicated in malaria and bilharzia, may also arise depending on the state of the surface water source, leading to additional human health hazards [13]. 

## 4. Overview of Steps Involved in Wastewater Treatment

**Figure 1:**
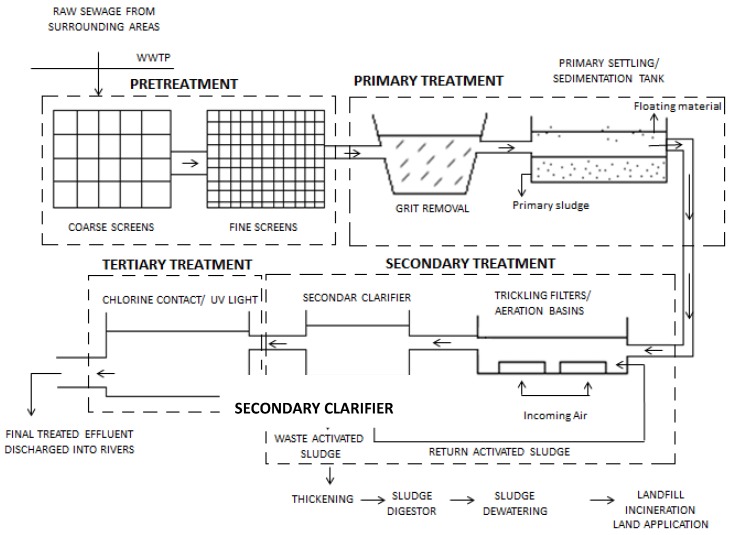
Overview of treatment stages within a wastewater treatment plant. Adapted from EPA [16] and UNEP [17].

The need to protect the current diminishing water resources has been constantly stressed, with increasing concerns about national water quality and maintenance of ecosystem health [14]. Initially, all wastewater used to be discharged directly into natural waterways, where a dilution effect would occur in conjunction with the degradation of organic matter by existing microorganisms. However, due to rising population numbers as well as an increase in the production of both domestic and industrial waste, the pollution of surrounding environments and consequent deterioration of public health has escalated [15]. This resulted in an increased need for the introduction of WWTPs that would aid and accelerate the purification process prior to discharge into any natural waterway. In addition, provided these plants operate efficiently, the treated wastewater effluent and sludge produced could serve as a valuable resource when reused safely. The overall wastewater treatment process can be broken down into four main stages namely the preliminary, primary, secondary and tertiary stages (Figure 1). 

### 4.1. Pretreatment

The first stage of treatment involves the use of screens to remove larger debris such as paper, plastic or any other foreign material which may damage downstream plant equipment. This is followed by further removal of grit and silt. In addition, the screened materials are often hazardous and must be safely disposed of to prevent fly breeding, excessive odors or downstream hazardous effects to public and environmental health. One such suitable disposable method is deposition in trenches covered with soil. In addition, the incineration of solids prior to burial is often preferred [5]. Excess grit such as sand, silt and stones can cause severe operational problems, affecting a range of subsequent treatment steps, ultimately causing severe pump blockages. Grit removal is therefore essential to protect mechanical equipment and pumps from abrasion and to reduce blockages. In addition measuring daily flows within a plant to ensure the maintenance of functional capacity is imperative to producing effluent of sufficient quality [18]. 

### 4.2. Primary Treatment

The main purpose of primary treatment is to reduce any settleable solids, as well as oils, grease, fats, sand and grit within the wastewater via settling and sedimentation processes. The steps involved in primary treatment are entirely mechanical and by means of filtration and sedimentation [18]. After initial screening to remove larger debris, wastewater still contains dissolved organic and inorganic constituents as well as suspended solids which are removed via the process of primary settling, sedimentation, chemical coagulation or filtration. This allows for separation of the solid and liquid phases in the wastewater by removing those settled organic solids as well as any floating materials such as fats, oil and grease. 

**Table 1:** Overview of various secondary treatment options available.

Treatment	Design criteria	Effluent quality	Advantages	Disadvantages	Ref.
**WASTE STABILISATION PONDS**					
**Anaerobic ponds**	2–5 m deep, pH usually below 6.5; less surface area; covered either by gravel, plants, steel, and plastic. Loaded at high rates to prevent inlet of any oxygen	BOD Removal of 60%–85%	Low cost, little excess sludge produced, Small pond volume needed; Low nutrient requirements; Low operating costs; no electricity required; Methane by-product	Requires more land; Long start-up period; Post treatment always required, can produce an unpleasant odour; Requires sludge removal more often; Operates optimally at warmer temperatures (>25 °C)	[10,19]
**Facultative ponds**	Shallow—1–3 m deep; Length to breadth ratio should be a minimum of 2:1; lined with compact clay (minimum thickness 0.3 m) or polyethylene; formation of two layers—aerobic at surface and anaerobic at bottom	BOD removal of 70%–85%	Efficient BOD reduction; Nutrient reduction by aerobic and anaerobic bacterial processes as well as by surrounding plants; Natural aeration of the upper layer via movement of air; Low energy consumption	Significant space requirements; Efficiency is strongly affected by environmental factors; continuous maintenance required	[10]
**Maturation ponds** **(polishing ponds)**	Shallow—0.9–1 m deep; allows for light penetration; completely aerobic; high pH and high concentration of dissolved oxygen due to algal activity; little biological stratification; size and number depends on required effluent pathogen concentration	Little BOD removal because most has been removed in previous stages	Removes excess nutrients and pathogens such as faecal coliforms	Small BOD removal; additional costs; additional land requirements	[10]
**SUSPENDED GROWTH SYSTEMS**					
**Activated sludge**	oxygen supplied for initial sludge decomposition and provide agitation to promote flocculation; 85% sludge removed whilst 15% recirculated	BOD removal of 90%–98%	Production of high quality effluent; reasonable operational and maintenance costs	High capital costs; high energy consumption; regular monitoring required; back washing needed	[20]
**Batch reactor**	Equalization, biological treatment and secondary clarification are performed in a single reactor vessel using a timed control sequence; aeration may be provided by bubble diffusers/floating aerators	BOD removal of 89%–98%	Initial capital cost savings; all processes carried out in a single reactor vessel; timed cycles; requires limited land; equalization of processes	Higher level of sophistication and maintenance required as timing must be controlled; may discharge settled or floating sludge; clogging of aeration devices; requires oversized outfalls as effluent discharge is timed	[21,22]
**SUSPENDED GROWTH SYSTEMS**					
**Aerated lagoons**	Should be lined with clay or some natural source, 1.8–6 m depth, 10–30 day retention time, oxygen supplied by additional mechanical means	BOD removal of up to 95%	Low cost, low maintenance and energy requirements, can be well integrated into surrounding landscapes, reliable treatment even at high loads	Nutrient removal is less efficient due to short retention times	[23,24]
**FIXED FILM SYSTEMS**					
**Conventional biofilters (trickling filters)**	Bed with supportive media such as stones, plastic, wood; 0.9–2.4 m deep; oxygen supplied via natural flow of air	BOD Removal of between 80%–90%	Low land requirement Moderate level of skill required for operation and maintenance Suitable for small to medium communities	Accumulation of excess biomass will affect performance; high level of clogging thus regular backwashing is required; if suddenly shut down–anaerobic conditions result in reduced effluent quality; odour and snail problems	[25,26]
**Rotating biological contactors**			High contact time; high effluent quality; resistant to shock hydraulic or organic loading; short contact periods; large active surface area; silent; low sludge production; easy transfer of oxygen from air	Continuous power supply required; oxygen may be a limiting substrate	[27]
**Biological aerated filters**	Consists of a reactor container, media for supporting biofilm growth, influent distribution and effluent collection system;Optimal conditions—pH 6.5–7.5 with mixing; Media should be chemically stable, high surface area and low weight e.g., sunken clay, floating polystyrene beads	High nutrient removal (80%–100%)	Environmental factors such as pH, temperature will aid microbial growth; high removal efficiencies; can combine ammonia oxidation and solids removal in a single unit	Media may become clogged due to biomass growth and accumulation—may create resistance to air and flow of liquid; regular back washing is required to remove excess biomass and particles	[28,29]

Wastewater enters a sedimentation tank, where the flow rate gradually slows down, enabling the wastewater to sit in these settling tanks which have been designed to hold the wastewater for several hours. During this time, most of the heavy solids fall to the bottom of the tank forming primary sludge thereby reducing the suspended solid content of the wastewater. In addition, any surface floating materials is usually siphoned off [15]. 

### 4.3. Secondary Treatment

Following primary treatment, wastewater flows into the next stage whereby remaining suspended solids are decomposed and the microbial load is greatly reduced. A variety of secondary treatment options are available (Table 1) which are classified into three main categories, namely, wastewater stabilization ponds, suspended growth systems or fixed film systems. This step results in organic matter removal of approximately 90% [16]. Wastewater stabilization ponds may be constructed either singularly or in parallel with the number of ponds increasing as the volume of waste being processed by the plant increases. These ponds are classified by the type of bacteria responsible for the decomposition process as well as the duration for which the waste will remain in the pond [7]. Suspended growth systems are generally applied to smaller communities and consist of three main types: activated sludge, sequential batch reactor and aerated lagoons whilst fixed film systems involve the passage of raw wastewater onto a filter medium in which bacteria can attach, build up and accumulate in biomass which is subsequently removed [16]. 

### 4.4. Disinfection and Tertiary Treatment Processes

Tertiary treatment generally follows secondary treatment and aids the removal of those wastewater constituents and pathogenic microorganisms such as faecal coliforms, streptococci, S*almonella* sp. and enteric viruses that are not removed by previous treatments [16]. Disinfection or tertiary treatment may be divided into three main treatment types namely; chemical, physical and irradiation. Physical treatments generally involve one or a combination of treatments such as rapid sand filtration, additional nutrient removal or carbon adsorption which is employed prior to chlorination to remove any remaining suspended solids as well as reduce the amount of nitrates, phosphates and soluble organic matter present [30]. Following this, disinfection by chemical and irradiation may occur and generally involves one or a combination of treatments involving chlorination and ultraviolet light exposure or ozonation, the choice of which depends solely on the incoming effluent quality, ease and cost of installation, maintenance and operation as well as effects on flora, fauna and recreational users from final effluent re-use and disposal into respective receiving water bodies [21]. 

#### 4.4.1. Disinfection

##### Chlorination

Chemical oxidation processes include the use of ozone, hydrogen peroxide and chlorine which may be applied in various forms such as pure chlorine, chlorine dioxide or chlorine compounds such as calcium or sodium hypochlorite. The major factors that need to be taken into consideration when evaluating the performance of chemical disinfectants are contact time, efficiency of mixing, concentration of chemicals used, residual remaining, pH and the concentration of interfering substances which may reduce the effectiveness of the disinfectant [31]. Chlorine is the commonly used method for disinfection of surface and groundwater sources, reacting with any form of organic matter that may be present in previously treated effluent. Chlorine gas is a strong oxidant that is most commonly used in larger treatment plants since it is more cost effective than other methods of tertiary treatment as well as allowing for easy and accurate application [32]. Chlorine dioxide is one of the most economical methods available and is a powerful oxidant that is capable of oxidising iron and manganese as well as removing any colour components in the effluent. Calcium hypochloride is available as granules, powder and tablets whilst sodium hypochlorite, also known as household bleach is a 13% solution of chlorine which is equivalent to 10%–12.2% available chlorine. This method however is extremely unstable and deteriorates rapidly. When elemental chlorine comes into contact with water, it is hydrolysed to hypochlorous acid (HOCl) and hypochlorite (-OCl), with HOCl being one of the strongest disinfecting agents. In addition, chlorine also reacts with ammonia to produce a range of mono- and dichloramines which serve as less potent disinfectants [33]:

NH_3_ + HOCl→**NH_2_CI**+H_2_O (monochloramine)

NH_2_C1 + HOC1→**NHC1_2_** + H_2_O (dichloramine)

NHCI_2_ + HOC1→**NCl_3_** + H_2_O (nitrogen trichloride)

However, one of the major disadvantages associated with chlorination is the production of toxic byproducts such as trichloromethanes and other chloramines which cause severe harmful effects on the receiving water bodies into which they are discharged [34].

##### Ultraviolet Light

Ultraviolet light has been increasingly used as a means of disinfection due to reduced safety hazards and decreased environmental toxicity. Being a physical form of disinfection, it eliminates the need to handle, transport or store toxic or corrosive chemicals. In addition, the potential to generate disinfectant byproducts is greatly reduced thereby eliminating any residual effect to human or aquatic life [34]. UV disinfection involves the use of electromagnetic energy from a mercury arc lamp to irradiate and disinfect wastewater effluent. The lamp itself should be routinely cleaned due to the large amount of interference from chemical components present in the wastewater being treated. The effectiveness of this disinfection method depends on the dose received as well as achieving an optimal wavelength in the range of 250–270 nm. In addition, a range of factors have to be taken into consideration such as effluent quality, UV light intensity, path length from the lamp to the respective pathogenic microorganisms as well as exposure time [31]. Disinfection generally occurs by inactivation of microbial cells through the formation of thymine dimers thereby affecting cell replication and the ability to infect a host. The UV light penetrates the cell wall of exposed microorganisms, ultimately damaging their genetic material and preventing survival. However when UV is applied at lower doses, microorganisms tend to reverse the damage through their own cellular repair mechanisms such as photoreactivation, nucleotide excision repair (dark repair) or recombination repair [33].

##### Ozonation

Ozone is a highly reactive, unstable gas that is generally used as a disinfectant and does not leave any residual behind, reacting with any organic matter present within the wastewater:

O_2_ + energy → O + O, then O + O_2_ → O_3_ (1)

Due to its unstable nature, it must often be generated onsite by the passage of oxygen through a high voltage electric field. The required ozone dosage is dependent on a range of factors, the most important being the type of effluent being treated. Previous studies have shown ozone requirements ranging between a few mg/L to greater than 10 mg/L for primary effluent [35]. Ozone is generally used as it results in the elimination of any odours, does not result in any residual compounds, can be easily generated from air thus resulting in the process being entirely dependent on the available power source. However, the major disadvantages include the high costs involved [36]. 

#### 4.4.2. Tertiary Treatment

##### Nutrient Removal

Tertiary treatments involving nutrient removal are often referred to as advanced methods of wastewater treatment and usually occur after or in conjunction with conventional biological secondary treatment to aid both nitrogen and phosphorous removal from wastewater. Generally these methods may include some form of physical or chemical techniques such as flocculation, precipitation or membrane filtration. Two such commonly used techniques include Biological Nutrient Removal and Enhanced Nutrient Removal which serves as a modification of the suspended growth treatment systems, achieving nitrogen and phosphorous removals of 8–10 mg/L; 1–3 mg/L and 3 mg/L; 0.3 mg/L per respective process [37]. Nitrogen is generally present in wastewater in the form of ammonia and is usually not removed by prior conventional secondary treatment processes. Therefore, advanced treatment methods successfully aid in the conversion of ammonia and other organic forms via nitrification and denitrification to non-toxic nitrate and subsequently nitrogen gas. Generally secondary biological treatment processes achieve phosphorous removal rates of less than 20%, requiring the need for additional removal methods. Physical precipitation such as filtration techniques as well as chemical precipitation such as flocculation after lime or alum addition may be used which aids in achieving phosphorous reduction rates of up to 95% [33]. 

##### Filtration

Membrane filtration technology is one such advanced method that has been implemented in removing micropollutants and can be divided into different categories depending of the type and size of contaminants to be removed. Media-coated filters contain one or more layers of an inert media such as sand or gravel and traps suspended particles either within pore spaces or through adherence to particle surfaces within the media-coated membrane. Pressure-driven membrane processes include a range of filtration types, namely, microfiltration, ultrafiltration, nanofiltration and reverse osmosis which are differentiated by pore size and which can be used to remove a range of residual micro pollutants after disinfection [38].

##### Activated Carbon

Activated carbon treatment is most often applied to treated industrial wastewater that will either be reused or which needs to meet stringent guidelines prior to discharge into any receiving watershed [39]. This advanced method has been shown to be effective in removing a range of soluble organic and inorganic compounds such as nitrates, heavy metals and a range of pharmaceuticals as water is passed over a bed of activated carbon granules. Adsorption occurs via adherence of pollutants to carbon particles after thermal activation [40]. Removal rates are however, affected by the composition of treated waste as the amount of organic matter present may reduce adsorption sites available [41]. 

## 5. Methods of Effluent Disposal

The type of treatment chosen per plant will depend solely on the incoming waste as well as where the treated effluent will be discharged. The discharge of waste is grouped into two main categories, based on the nature of the waste source namely, point source and diffuse source. Diffuse sources are initially discharged as a point source, after which it migrates towards the water resource and has a diffuse impact [42]. The actual destination of discharges is important because it largely determines the extent and nature of the impact. In addition, the waste volume may also disturb natural cycles in receiving water bodies such as rivers ultimately affecting not only the water quality but also water flow. Larger municipalities located near coastlines may opt to discharge treated effluent directly into the ocean whereby oceanic processes can be used to reduce effluent contaminant concentrations to the required recreational guidelines and to comply with environmental standards. In addition, due to the constant changing physical conditions along South African coastlines, responsible disposal of wastewater to the marine environment is allowed due to the reduction of contaminant concentrations brought about by the initial dilution of effluent, dispersion of the effluent plume and decay of microorganisms. Furthermore, depending on the type of effluent, surrounding areas, state of the coastline and degree of dilution that can be achieved after oceanic discharge, the wastewater may require different stages of pre-treatment prior to discharge (Table 2). Within the eThekwini Municipality in South Africa itself, two major wastewater treatment works, namely The Central Works and Southern Works both discharge effluent into the Indian Ocean. Within these plants, initial influent is subjected to conventional screening, de-gritting and primary sedimentation followed by subsequent discharge to the sea via outfall pipes.

**Table 2:** Overview of treatment requirements for selected effluent discharges.

Destination	Preliminary	Primary	Secondary	Tertiary
Irrigation				
Produce Eaten Raw	YES	YES	YES	YES
Other Produce	YES	YES	YES	NO
GroundWater	YES	YES	YES	YES
Surface Waters	YES	YES	YES	NO
Sea Outfalls	YES	YES	YES	NO

Note: Adapted from Wastewater Treatment Guidance Manuel—Syrian Lebanese Higher Council [32].

## 6. Commonly Detected Microbial Indicators in Treated Wastewater Effluent

The World Health Organisation estimates that globally, approximately 1.1 billion people consume unsafe water with approximately 88% of diarrhoeal diseases and 1.7 million deaths worldwide being attributable to unsafe water, sanitation and hygiene [43]. Microbiological examination and monitoring is commonly used worldwide to ensure the safety of a range of water sources whereby contamination with human and animal excreta could pose serious risks. Many potential pathogens (Table 3) could be associated with contaminated water however; it is both time consuming and expensive to test for all possible pathogens present. Hence, representative indicators associated with human and animal contamination are used as a means to detect such pollution [44].

**Table 3:** Microbial indicators and pathogenic organisms associated with waterborne diseases and common sources of contamination.

Microorganisms		Diseases	Source	Numbers *
Bacteria	*Salmonella enterica* subsp. *enterica* serovar Typhi	Thyphoid fever	Human faeces	0.2–8,000
	*Salmonella enterica* subsp. *enterica* serovar Paratyphi	Paratyphoid fever	Human faeces	
	*Salmonella enterica* subsp. *enterica* serovar Enteritidis and *Salmonella enterica* subsp. *enterica* serovar Typhimurium	Salmonellosis/gastroenteritis	Human/animal	
	*Shigella* sp. *(Shigella dysenteriae*, *Shigella flexneri*, *Shigella boydii*, *Shigella sonnei)*	Dysentery	Human faeces	0.1–1,000
	*Vibrio* *cholera*	Cholera	Human faeces	
	*Vibrio* *parahaemolyticus*	Gastroenteritis	Human/animal	
	*E. coli* (*E. coli* O:148; O:157; O:124)	Gastroenteritis	Human faeces	10^6^–10^7^
	*Campylobacter* sp.	Gastroenteritis	Human/animal	10^4^–10^5^
	*Clostridium* *perfringens*		Human/animal	6 × 10^4^–8 × 10^4^
	Faecal streptococci		Human/animal	4.7 × 10^3^–4 × 10^5^
	Enterococci		Human/animal	
Viruses	Poliovirus	Poliomyelitis	Human faeces	180–500,000
	Rotavirus	Diahorrea, vomiting	Human faeces	400–85,000
	Adenovirus	Gastroenteritis	Human faeces	
	Norwalk virus	Diahorrea, vomiting	Human faeces	
	Hepatitis A Virus	Hepatitis	Human faeces	
Protozoa	Cryptosporidium parvum	Diahorrea		0.1–39
	Entamoeba histolytica	Amoeba dysentery		0.4
	Giardia lamblia cysts	Diahorrea		12.5–20,000

Note: * Numbers of infectious particles in raw sewage. Adapted from Ashbolt and Grabow *et al*. [45,46].

### 6.1. Total and Faecal Coliforms

Total coliforms have been defined as all those aerobic or facultative anaerobic, gram-negative, non-spore-forming, oxidase-negative, rod-shaped bacteria which have the ability to ferment lactose with gas and acid formation within 48 h at 35 °C whilst faecal coliforms have been defined as those coliforms which can proliferate at an elevated temperature of 44.5 °C [43]. The total coliform group includes those microorganisms that can survive and proliferate within the water environment and includes several species of the *Enterobacteriaceae* family, belonging to the genera *Escherichia*, *Citrobacter*, *Klebsiella* and *Enterobacter*. These bacteria live in the human and animal intestine, but some *Citrobacter*, *Enterobacter* and *Klebsiella* species are also found in terrestrial environments and are therefore not necessarily indicators of faecal pollution. They have also been found to occur in both sewage and natural water sources with some of these bacteria being excreted in the faeces of humans and animals. In addition, they are far more sensitive to disinfection than enteric viruses and protozoa and thus should be absent immediately after disinfection indicating that their presence serves as an indication of inadequate wastewater treatment [45]. Faecal coliforms include *E. coli* and some *Klebsiella* species, such as *K. oxytoca* and *K. pneumonia*. Whilst *E. coli* serves as a true indicator of faecal pollution, its survival in the environment is limited. Faecal coliforms are therefore much better indicators of faecal pollution than total coliforms although some false positives may occur due to the ubiquitous nature of *Klebsiella* sp. [47].

### 6.2. E. coli

*E. coli* is commonly regarded as one of first microorganisms of choice in water quality monitoring programs and serves as the primary indicator for water contaminated with faecal matter due to their prevalence in the gut of warm-blooded animals as well as high numbers excreted in both human and animal faeces [46]. Six major pathogenic classes that have been identified namely, enterotoxigenic *E. coli* (ETEC), enterohaemorrhagic or shiga-toxin poducing *E. coli* (EHEC), enteroinvasive *E. coli* (EIEC), enterpathogenic *E. coli* (EPEC), enteroadherent-aggregative *E. coli* (EA-AggEC) and diffuse adherent *E. coli* (DAEC) [48,49]. Enteropathogenic *E. coli* (EPEC) have been primarily associated with outbreaks of infantile gastroenteritis whilst enteroinvasive *E. coli* (EIEC) are known to produce dysentery by a mechanism similar to *Shigella* sp. causing severe bloody diarrhoea whilst enterotoxigenic *E. coli* (ETEC) are known to possess a heat-labile enterotoxin similar to the cholera toxin [4]. The most important is EHEC which includes the -O111 and -O157 serogroups all of which produce a shiga-like toxin resulting in mild diarrhoea to haemorrhagic colitis [50]. EHEC has been implicated in a range of foodborne-related outbreaks since 1983 with one of the largest European outbreaks occurring recently in 2011, caused by the Shiga toxin-producing *E. coli* (STEC) O104:H4. Numerous studies revealed a unique combination of virulence factors from two distinct *E. coli* classes, namely, enteroaggregative *E coli* (EAEC) and STEC which contributed to its pathogenic nature [51,52]. Despite being a foodborne pathogen, numerous studies have implicated contamination from infected individuals. 

### 6.3. Faecal Streptococci and Enterococci

Faecal streptococci belong to the traditional indicator group of fecal pollution and are defined as gram-positive, catalase-negative, non-spore-forming cocci that grow at 35 °C. Enterococci are gram-positive, facultative anaerobic bacteria which possess the ability to grow in the presence of 6.5% NaCl at a high temperature of 45 °C. These bacteria grow in media with azide, since they do not have a respiratory chain. They are found in both, human and animal intestines but some species also inhabit terrestrial environments and animal products such as milk, cheese and meat [53]. Enterococci form a sub-group of the larger faecal streptococci group and consist of the species *Enterococcus faecalis*, *E. faecium*, *E. durans* and *E. hirae* which are well known for their association to faecal pollution. Members of this group are typically excreted in the faeces of humans and other warm-blooded animals and are present in large numbers in water environments polluted by sewage or human and animal waste. Several studies that examined both human and animal faeces have concluded that both *E. faecalis* and *E. faecium* are responsible for the majority of human infections and are most frequently found in human faeces whilst *E. avium*, *E. cecorum*, *E. durans*, *E. gallinarum* and *E. hirae* are most commonly found in animal faeces [48]. However, other studies have also shown varying ratios of the presence of *E. gallinarium* in human faeces whilst *E. faecium* and *E. faecalis* had been isolated in a range of animal faeces as well, indicating no single species of *Enterococcus* serves as a reliable indicator of human faecal contamination [53,54]. In addition to being tolerant to sodium chloride and alkaline pH levels, they do not multiply in water environments making them suitable indicators of faecal contamination [55]. They are also known to survive for longer periods as when compared to *E. coli* and are more resistant to chlorination making them suitable indicators of inefficient disinfection processes [4]. The intestinal enterococci group has been used as an index of faecal pollution with additional studies showing the numbers of intestinal enterococci in human faeces being about an order of magnitude lower than that of *E. coli* [55]. 

### 6.4. Salmonella *sp.*

*Salmonella* sp. belong to the family Enterobacteriaceae and are defined as motile, gram-negative bacilli that are oxidase negative, catalase positive and which utilize citrate as a sole carbon source. *Salmonella* sp. are generally transmitted via the faecal-oral route, with infections being characterised by mild to full blown diarrhoea, nausea, vomiting, septicaemia, typhoid and enteric fever [43]. Host specific human infections are grouped into two main categories namely (1): typhoid and paratyphoid and (2): gastroenteritis. In addition, other strains such as *Salmonella enterica* subsp. *enterica* serovar Typhimurium and *Salmonella enterica* subsp. *enterica* serovar Enteritidis are known to infect both humans and a wide range of animals such as poultry, cows, pigs, sheep, birds and even reptiles [56]. The main habitat of *Salmonella* sp. is the intestinal tract of both humans and animals. These pathogens are not known to multiply significantly in the environment, however previous studies have shown prolonged periods of survival in both water and soil environments, provided external temperature, humidity and pH conditions are favourable. In addition, infected individuals are known to carry the bacteria for extended periods of time without any signs of infection. Previous studies have shown that *Salmonella* sp. can survive wastewater treatment processes thereby entering surface water sources and serving as a source of contamination to filter feeders such as shellfish, ultimately entering the food chain [55]. 

### 6.5. Shigella *sp.*

*Shigella sp*. are defined as gram-negative, non-spore forming, non-motile, catalase positive, oxidase negative, rod-like members of the family *Enterobacteriaceae* and are characterized based on the presence of their O-antigens [55]. Approximately 164.7 million cases of waterborne diseases within developing regions can be attributed to *Shigella*-related infections annually with approximately 61% of all shigellosis-related deaths occurring in children under five years. *Shigella* sp. is generally an inhabitant of the intestinal tract of humans and is often spread by faecal contaminated water, food sources or by direct contact with infected individuals. These bacteria can survive for months in the intestine of chronic patients. Survival outside the human intestine is limited but bacteria appear to be able to survive in waters in a viable-but-non-culturable state. In addition, *Shigella* sp. is known to survive for extended periods of up to 6 months at room temperature, thus aiding their transmission within the water environment. There are four important species which have been implicated in numerous global *Shigella*-related epidemics namely, *S. dysenteriae* (15 serotypes), *S. flexneri* (8 serotypes), *S. boydii* (19 serotypes) and *S. sonnei* (1 serotype). *Shigella* sp. infections are characterised by an extremely low infectious dose, ranging between 10–100 infectious particles with symptoms consisting of abdominal cramps, fever and watery diarrhoea after 24–72 h of infection [56]. In addition, *S. dysenteriae*, *S. sonnei and S. flexneri* are known to produce the cytotoxic Shiga-toxin which inhibits mammalian protein synthesis. As found for *Vibrio cholerae*, genes for toxin production can be transmitted horizontally. The production of a lipopolysaccharide endotoxin is also produced together with a range of plasmids coding for a host of virulence genes encoding for the production of adhesins aiding attachment, invasion plasmid antigens and factors aiding transport or processing functions [55].

### 6.6. Vibrio *sp.*

*Vibrio* sp. are motile, oxidase positive, non-spore-forming, gram-negative rods with a single flagellum defined as being facultative anaerobes. They are frequently found in marine and estuarine environments, usually transmitted via the faecal–oral route with infections generally caused by ingestion of faecal-contaminated water and food [55]. There are a number of pathogenic species implicated in gasteroenteritis, including *V. cholerae*, *V. fluvialis*, *V. furnissii*, *V. hollisae*, *V. mimicus*, *V. parahaemolyticus* and *V. vulnificus* with *V. cholerae* being the major pathogen of concern within the water environment. Whilst non-toxigenic *V. cholerae* is widely distributed in water environments, toxigenic strains are not distributed as widely, with only the O1 and O139 serovars being known to cause the related-cholera symptoms due to the production of the cholera exotoxin. Symptoms of infected individuals include changes in ionic fluxes across the intestinal mucosa leading to severe water and electrolyte loss [4,57].

### 6.7. Coliphages

Coliphages are defined as bacteriophages or viruses that utilise bacteria such as *E. coli* and related species such as *Salmonella* sp. as a host for replication. They are often used in water quality assessments and are divided into two major groups namely, somatic and F-RNA coliphages based on their route of infection. Coliphages have shown to exhibit characteristics that meet the basic requirements of surrogates for enteric viruses, such that they should be present in water environments whenever enteric viruses are present, be present in the same or higher numbers than viruses, be similarly resistant as viruses to purification and disinfection processes, be specific for fecal contamination, non-pathogenic, not be able to multiply in water environments and be detectable by simple, rapid and inexpensive methods [58,59]. Furthermore, coliphages share many similar properties with human enteric viruses such as structure, composition, morphology, size and site of replication, thus further reinforcing their valuable nature as models for enteric viral detection. In addition, the use of simple, inexpensive and rapid techniques have further contributed to their suitability as ideal indicators that can be used in conjunction with bacterial indicators to determine water quality [48]. Structural and morphological analyses indicate the presence of a nucleic acid molecule surrounded by a protein coat also known as the capsid. They are known to both replicate in the gastrointestinal tract of humans and warm-blooded animals. Generally, somatic coliphages have been found to outnumber F-RNA coliphages in water environments by a factor of approximately 5 and cytopathogenic human viruses by a factor of about 500 [4]. 

#### 6.7.1. Somatic Coliphages

Somatic coliphages consist of a wide range of phages belonging to members of the families—Myoviridae, Siphoviridae, Podoviridae and Microviridae with a vast range of morphological types. These phages possess the ability to infect hosts such as *E. coli* and other closely related members of the Enterobacteriaceae family by attaching to receptors permanently located on the cell wall of hosts. They replicate more frequently in the gastrointestinal tract of warm-blooded animals but have also been known to replicate in water environments [46]. 

#### 6.7.2. Male Specific F-RNA Coliphages

F-RNA coliphages are defined as single stranded RNA phages that are morphologically similar to that of picornaviruses. These phages possess an icosahedral capsid and belong to the family Leviviridae which have further been divided into four serological groups allowing for source contaminant identification. Serogroups I and IV are known to be excreted in animal faeces whilst serogroup III are known to be excreted in human faeces [43]. Contaminant source identification together with their physical structure, composition and morphology have allowed phages to serve as a means to distinguish between faecal pollution of human and animal origin as well as an ideal indicator of the presence of human enteric viruses. In addition, numerous studies have revealed that F-RNA phage counts outnumber enteric viruses by a factor of approximately 100 in wastewater and raw water sources. However, one of the major disadvantages is that currently utilised F-RNA coliphage detection schemes are not as simple due to the requirement of adequate host preparation prior to detection. F-RNA coliphages initiate infection by attaching to fertility fimbriae which are produced by hosts in the exponential growth phase at temperatures above 30 °C, indicating the need for timeous preparation of host cultures [4]. 

#### 6.7.3. Phages that Infect *Bacteroides fragilis*

Considerable attention has been given to *Bacteroides fragilis* as an indicator of water quality as they are found solely in human faeces and show resistance to a range of chlorine disinfection steps when compared to other pathogens such as poliovirus, rotavirus, certain coliphages, *E. coli* and *Streptococcus faecalis*. Two groups of *B. fragilis* phages are used as indicators in water quality assessment. They differ by their respective bacterial host strains, *Bacteroides* HSP40 and RYC2056 which inhabit the gasterointestinal tract. The first group belongs to the family Siphoviridae and are restricted solely to the human gasterointestinal tract and hence their detection within the environment provides a strong indication of human faecal contamination. The second group however includes a wider range of phages which are detected in both human and animal faeces [43]. However, as with all indicators, one of the major disadvantages is that HSP40 phages are excreted by approximately 10%–20% of humans in certain parts of the world and hence occur in relatively low numbers in sewage, polluted water environments and drinking water sources indicating that their absence does not confirm the absence of other pathogens such as enteric viruses. In addition, detection methods have proven to be more complex and expensive due to the need for antibiotics and anaerobic environments, than those for somatic and F-RNA coliphages [46].

## 7. Current Guidelines for Treated Effluent

Various guidelines and water quality criteria have been set in both local and international committees, however, due to the vast differences in methodology and development; these values tend to differ greatly. Whilst some guidelines tend to exhibit the maximum concentration of a particular contaminant, others attempt to define the ideal concentration thus leading to confusion. In addition, these guidelines need to be flexible and adapted to suit local, regional and national scenarios by taking the current socio-economic and environmental conditions into consideration [60]. This should be followed by subsequent translation of guidelines into legally enforceable national standards by Government. Despite an outdated guideline for treated effluent being discharged into any catchment or river (Table 4), a South African Green Drop Certification Program was recently started by the Department of Water Affairs in an attempt to regularly monitor and improve the wastewater sector. This program allows for local municipalities to generate information from data pertaining to the efficiency of their treatment plants as well as effluent characteristics, in order to monitor and report on their wastewater management systems. In addition, it provides an overview of required information allowing for improved trend monitoring and decision making as well as providing the public with access to relevant information regarding wastewater treatment within their regions [5]. 

**Table 4:** Currently used guidelines by the eThekwini Municipality (South Africa) for treated effluent being discharged into a receiving catchment.

Parameter	A	B
Colour/Odour/Taste	None	None
pH	5.5–9.5	5.5–7.5
Dissolved Oxygen (mg/L)	75% saturation	75% saturation
Faecal Coliforms (CFU/100 mL)	0	0
Temperature (°C)	35	25
Chemical Oxygen Demand (mg/L)	75	30
Electrical Conductivity (mS/m)	75	
Total Suspended Solids (mg/L)	90	10
Sodium Content (mg/L)	90	50
Soap/Oil/Grease (mg/L)	2.5	None
Residual Chlorine (mg/L)	0.1	0
Free/Saline Ammonia (mg/L)	1	1
Nitrate (mg/L)	None	1.5
Orthophosphate (mg/L)	1	1

Notes: Adapted from Government Gazette [61]. (A): Guidelines for effluent being discharged into any area other than that specified by B; (B): Guidelines for effluent being discharged into any catchment area/river or a tributary.

## 8. Conclusions

It is a well-known fact that man has dominated the planet for decades and with constantly increasing population numbers, hydrological variability and rapid urbanization coupled with the need for greater socio-economic development, man will continue to play an ever increasing dominant role. In addition, obtaining a global perspective of surface water quality has become increasingly difficult as different nations struggle with different environmental pressures, more so in developing countries where available resources are limited. One such visible example is the increasing volume and pressure on existing wastewater treatment plants together with surrounding inefficient hygiene practices and exacerbated nutrient and microbiological loads constantly entering receiving river systems and water supplies. Increased pressure on existing infrastructure coupled with the use of outdated guidelines for treated effluent has further compounded these issues. This has ultimately resulted, not only in an increase in waterborne diseases but also an increase in waterborne-disease-related deaths [13]. In addition, despite constant monitoring and the use of a range of microbial water quality indicators, the implementation of effective preventative strategies together with advanced wastewater treatment concepts incorporating a more sustainable approach is required to ensure both protection and sustainable use of limited and unreliable water resources [62]. Ensuring efficient surveillance and management of existing treatment plants coupled with guideline revision and monitoring compliance is imperative to preventing further risk of pollution to the environment and human health.

## References

[1] United Nations Water Global Annual Assessment of Sanitation and Drinking Water (GLAAS) (2012). The Challenge of Extending and Sustaining Services.

[2] UNICEF and World Health Organization (2012). Progress on Drinking Water and Sanitation. Joint Monitoring Programme for Water Supply and Sanitation.

[3] World Water Assessment Programme (WWAP) (2012). The United Nations World Water Development Report 4: Managing Water under Uncertainty and Risk.

[4] World Health Organization (2011). Guidelines for Drinking-Water Quality.

[5] Department of Water Affairs (2011). Green Drop Handbook, Version 1.

[6] (2009). CIDWT Decentralized Wastewater Glossary. http://www.onsiteconsortium.org/glossary.html.

[7] Mara D. (2004). Domestic Wastewater Treatment in Developing Communities.

[8] Hamdy A., Shatanawi M., Smadi H. (2005). Urban wastewater problems, risks and its potential use for irrigation. The use of non-conventional water resources. Bari: CIHEAM/EU-DG Res..

[9] Environmental Protection Administration, Taiwan Water: Domestic Wastewater and Pollution. http://www.epa.gov.tw/en/epashow.aspx?list=102&path=135&guid=c4b6ad0f-13e5-4259-be98-8356037dc862&lang=en-us.

[10] Department of Water Affairs and Forestry (1996). South African Water Quality Guidelines. Volume 1: Domestic Water Use.

[11] Rosenwinkel K.H., Austermann-Haun U., Meyer H., Jördening H.-J., Winter J. (2005). Industrial Wastewater Sources and Treatment Strategies. Environmental Biotechnology. Concepts and Applications.

[12] Wakelin S.A., Colloff M.J., Kookanal R.S. (2008). Effect of wastewater treatment plant effluent on microbial function and community structure in the sediment of a freshwater stream with variable seasonal flow. Appl. Environ. Microbiol..

[13] Coetzee M.A.S. (2003). Water Pollution in South Africa: Its Impact on Wetland Biota.

[14] Okoh A.I., Sibanda T., Gusha S.S. (2010). Inadequately treated wastewater as a source of human enteric viruses in the environment. Int. J. Environ. Res. Public Health..

[15] USEPA (2004). Primer for Municipal Wastewater Treatment Systems. http://www.epa.gov.owm.

[16] Environmental Protection Agency (1997). Wastewater Treatment Manuals—Primary, Secondary and Tertiary Treatment.

[17] United Nations Environmental Programme. Division of Technology, Industry and Economics (2012). Biosolids, Management and Environmentally Sound Approach for Managing Sewage Treatment Plant Sludge. http://www.unep.or.jp/ietc/publications/freshwater/fms1/2.asp.

[18] Sonune A., Ghate R. (2004). Developments in wastewater treatment methods. Desalination.

[19] Alexiou G.E., Mara D.D. (2003). Anaerobic wastewater stabilization ponds: A low cost contribution to a sustainable wastewater reuse cycle. Appl. Biochem. Biotechnol..

[20] Norton A., Björnberg S., Kibirige D., Raja A.B. Wastewater Treatment–Treatment Ponds. http://www.chemeng.lth.se/vvan01/Arkiv/Report_2012_Treatment_Ponds.pdf.

[21] USEPA (1999). Wastewater Technology Fact Sheet–Sequencing Batch Reactors.

[22] Mahvi A.H. (2008). Sequencing batch reactor: A promising technology in wastewater treatment. Iran. J. Environ. Health Sci. Eng..

[23] USEPA (2002). Wastewater Technology Fact Sheet–Aerated, Partial Mix Lagoons.

[24] FUCHS Clean Solutions (2011). Aerated Lagoons for the Treatment of Municipal Wastewater. http://www.fuchs-germany.com.

[25] Chaudhary D.S., Vigneswaran S., Ngo H., Shim W.G., Moon H. (2003). Biofilter in water and wastewater treatment. Korean J. Chem. Eng..

[26] USEPA (2000). Wastewater Technology Fact Sheet–Trickling Filters.

[27] Kadu P.A., Rao Y.R.M. (2012). A review of rotating biological contactors system. IJERA.

[28] Mendoza-Espinosa L., Stephenson T. (1999). A review of biological aerated filters (BAFs) for wastewater treatment. Environ. Eng. Sci..

[29] Asiedu K. (2001). Evaluating Biological Treatment Systems–Moving Bed Biofilm Reactor vs. Biological Aerated Filtration and Sulfide-induced Corrosion in Anaerobic Digester Gas Piping.

[30] SOPAC Small Scale Wastewater Treatment Plant Project. Report on Project Criteria, Guidelines and Technologies. http://ict.sopac.org/VirLib/TR0288.pdf.

[31] USEPA (1999). Wastewater Technology Fact Sheet–Chlorine Disinfection.

[32] (2004). University Curriculum Development for Decentralized Wastewater Management. http://www.onsiteconsortium.org/ed_curriculum/University/IV.%20B.%20Septic%20Tanks/uniseptictext.pdf.

[33] Syrian Lebanese Higher Council (2012). Wastewater Treatment Guidance Manuel–Integrated Coastal Management between Jbeil/Amsheet (Lebanon) and Latakia (Syria).

[34] Basu S., Page J., Wei I.W. (2007). UV disinfection of treated wastewater effluent: Influence of colour, reactivation and regrowth of coliform bacteria. Environ. Eng.: Appl. Res. Pract..

[35] Lazarova V., Savoye P., Janex M.L., Blatchley E.R., Pommepuy M. (1999). Advanced wastewater disinfection technologies: State of the art and perspectives. Water Sci. Technol..

[36] USEPA (1999). Wastewater Technology Fact Sheet–Ultraviolet Disinfection.

[37] Hartman P., Cleland J. (2007). Wastewater Treatment Performance and Cost Data to Support an Affordability Analysis for Water Quality Standards.

[38] Bolong N., Ismail A.F., Salim M.R., Matsuura T. (2009). A review of the effects of emerging contaminants in wastewater and options for their removal. Desalination.

[39] Cecen F., Aktas O. (2011). Activated Carbon for Water and Wastewater Treatment: Integration of Adsorption and Biological Treatment.

[40] USEPA (2000). United States. Wastewater Technology Fact Sheet–Granular Activated Carbon Adsorption and Regeneration.

[41] Minnesota Water Sustainability Framework (2011). Wastewater Treatment Best Practices. Wastewater Treatment Best Practices. http://wrc.umn.edu/prod/groups/cfans/@pub/@cfans/@wrc/documents/asset/cfans_asset_292046.pdf.

[42] Department of Water Affairs and Forestry (2003). Water Quality Management Series. Sub-Series No. MS11. Towards a Strategy for a Waste Discharge Charge System.

[43] (2008). Guidelines for Drinking-water Quality (Electronic Resource): Incorporating 1st and 2nd Addenda, Volume 1, Recommendations.

[44] Barrell R.A.E., Hunter P.R., Nichols G. (2000). Microbiological standards for water and their relationship to health risk. Commun. Dis. Public Health..

[45] Ashbolt N.J. (2004). Microbial contamination of drinking water and disease outcomes in developing regions. Toxicology.

[46] Grabow W. (2001). Bacteriophages: Update on application as models for viruses in water. Water Sa.

[47] Leclerc H., Mossel D.A.A., Edberg S.C., Struijk C.B. (2001). Advances in the bacteriology of the coliform group: Their suitability as markers of microbial water safety. Annu. Rev. Microbiol..

[48] NHMRC, NRMMC (2011). Australian Drinking Water Guidelines Paper 6 National Water Quality Management Strategy.

[49] Nataro J.P., Kaper J.B. (1998). Diarrhaegenic *Escherichia coli*. Clin. Microbiol. Rev..

[50] Rice E.W., Johnson C.H., Reasoner D.J. (1996). Detection of *Escherichia coli* O157:H7 in water from coliform enrichment cultures. Lett. Appl. Microbiol..

[51] Wu C.J., Hsueh P.R., Ko W.C. (2011). A new health threat in Europe: Shiga toxin—Producing *Escherichia coli* 0104:H4 infections. J. Microbiol. Immunol. Infect..

[52] Soon J.M., Seaman P., Baines R.N. (2013). *Escherichia coli* O104:H4 outbreak from sprouted seeds. Int. J. Hyg. Environ. Heal..

[53] Wheeler A.L., Hartel P.G., Godfrey D.G., Hill J.L., Segars W.I. (2002). Potential of *Enterococcus faecalis* as a human fecal indicator for microbial source tracking. J. Environ. Qual..

[54] Layton B.A., Walters S.P., Lam L.H., Boehm A.B. (2009). *Enterococcus* species distribution among human and animal hosts using multiplex PCR. J. Appl. Microbiol..

[55] Cabral J.P.S. (2010). Water microbiology. Bacterial pathogens and water. Int. J. Environ. Res. Public Health.

[56] Angulo F.J., Tippen S., Sharp D.J., Payne B.J., Collier C., Hill J.E., Barett T.J., Clark R.M., Geldrich E.E., Donnell H.D. (1997). A community waterborne outbreak of salmonellosis and the effectiveness of a boil water order. Amer. J. Public Health.

[57] World Health Organization (2002). Guidelines for Drinking-Water Quality.

[58] Kott Y. (1981). Viruses and bacteriophages. Sci. Total Environ..

[59] Grabow W.O.K. (1986). Indicator systems for assessment of the virological safety of treated drinking water. Water Sci. Technol..

[60] (2003). Guidelines for Safe Recreational Water Environments.

[61] Government Gazette (1984). No. 9225. Requirements for the Purification of Waste Water or Effluent. General and Special Standards.

[62] Tchobanoglous G., Burton F.L., Stensel H.D. (2002). Wastewater Engineering Treatment and Reuse.

